# Comment on “Oncological and Survival Endpoints in Cancer Cachexia Clinical Trials: Systematic Review” by Dajani et al.

**DOI:** 10.1002/jcsm.13842

**Published:** 2025-06-04

**Authors:** Shubham Kumar, Rachana Mehta, Ranjana Sah, Edward Mawejje

**Affiliations:** ^1^ Center for Global Health Research, Saveetha Medical College and Hospital Saveetha Institute of Medical and Technical Sciences, Saveetha University Chennai India; ^2^ Clinical Microbiology, RDC Manav Rachna International Institute of Research and Studies Faridabad India; ^3^ Department of Paediatrics Dr. D. Y. Patil Medical College Hospital and Research Centre, Dr. D. Y. Patil Vidyapeeth (Deemed‐to‐be‐University) Pimpri India; ^4^ Department of Public Health Dentistry Dr. D. Y. Patil Medical College Hospital and Research Centre, Dr. D. Y. Patil Vidyapeeth (Deemed‐to‐be‐University) Pimpri India; ^5^ School of Public Health Makerere University College of Health Sciences, Mulago Hill Kampala Uganda

Dear Editor,

We recently had the pleasure of reviewing the systematic review by Dajani et al. [1], titled ‘Oncological and Survival Endpoints in Cancer Cachexia Clinical Trials: Systematic Review’. This work, the sixth in the Cachexia Endpoints Series, impressively synthesizes data from 57 trials involving 9743 patients, offering a comprehensive overview of oncological endpoints in cancer cachexia (CC) research. We commend the authors for their meticulous approach, adhering to PRISMA guidelines and employing a robust methodology that includes a thorough literature search across MEDLINE, Embase and Cochrane databases from 1990 to 2023. Their findings that overall survival (OS) and adverse events (AEs) dominate as endpoints, yet yield few significant outcomes highlight critical gaps in the field. To further enrich this commendable effort, we respectfully propose a few contextual suggestions that could enhance its interpretative depth and guide future research, presented with appreciation for the authors' significant contribution.

Firstly, we appreciate the authors' detailed reporting of trial heterogeneity, spanning tumour types, stages and interventions. Building on this, we suggest exploring the influence of cachexia severity at baseline on oncological outcomes. Trials often vary in their definitions of cachexia, and stratifying results by severity (e.g., weight loss percentage or muscle mass depletion) could reveal whether more advanced cachexia modulates treatment effects on OS or progression‐free survival (PFS). This could provide nuanced insights into which patient subgroups might benefit most from anticachexia interventions.

Secondly, while the authors adeptly note the lack of statistical power and multiple testing adjustments, we propose examining the role of treatment sequencing as a contextual factor. Many trials included patients on concurrent anti‐cancer therapies, yet the timing and sequence of these treatments relative to cachexia interventions remain underexplored. Analysing whether synchronous versus sequential administration impacts endpoints like treatment completion (TC) or AEs could elucidate potential synergistic or antagonistic effects, offering a practical lens for clinical trial design.

Thirdly, we commend the focus on OS and AEs as relevant endpoints, but suggest incorporating a patient‐centric perspective by assessing the interplay between oncological outcomes and cachexia‐related symptom burden. For instance, linking OS or TC to changes in fatigue or nutritional status could bridge oncological and quality‐of‐life domains, reflecting the holistic impact of cachexia on patients. This integrative approach might resonate with regulatory bodies prioritizing patient‐reported outcomes alongside survival metrics.

We propose considering the socioeconomic context of trial populations, an aspect not yet emphasized. Variations in access to nutritional support or anti‐cancer therapies across regions (e.g., high‐ vs. low‐income settings) could influence endpoint outcomes, particularly OS and AEs. A subgroup analysis or discussion of this factor might contextualize the generalizability of findings and inform equitable intervention strategies.

Dajani et al. have delivered a pivotal resource that illuminates the landscape of oncological endpoints in CC trials. We sincerely praise their rigorous scholarship and encourage the exploration of cachexia severity, treatment sequencing, symptom interplay and socioeconomic factors. These additions could further elevate this work's already substantial impact on future research and clinical practice.

## Conflicts of Interest

The authors declare no conflicts of interest.
